# Safety of Vaccination within First Year of Life—The Experience of One General Medicine Center

**DOI:** 10.3390/children10010104

**Published:** 2023-01-04

**Authors:** Claudia Felicia Pop, Petronela Coblisan, Ligia Capalna, Paraschiva Cherecheș Panța, Anca Dana Buzoianu, Ioana Corina Bocsan

**Affiliations:** 1 Department of Mother and Child, Nursing Discipline, Faculty of Medicine, University of Medicine and Pharmacy Ïuliu Hatíeganu”, 400124 Cluj-Napoca, Romania; 2 Intensive Care Unit, “Octavian Fodor” Institute of Gastroenterology and Hepatology, 400124 Cluj-Napoca, Romania; 3 Department of Mother and Child, 3rd Pediatric Discipline, Faculty of Medicine, University of Medicine and Pharmacy Ïuliu Hatíeganu”, 400124 Cluj-Napoca, Romania; 4 Department of Pharmacology, Toxicology and Clinical Pharmacology, Iuliu Haţieganu University of Medicine and Pharmacy, 400337 Cluj-Napoca, Romania

**Keywords:** vaccine, vaccine hesitancy, vaccine safety

## Abstract

Vaccines represent an important preventive strategy in paediatric populations, but the rate of vaccination has been constantly declining in the last decade. Concerns about vaccines’ safety represent one of the main causes of vaccine hesitancy among parents. The aim of this study was the analysis of the immediate adverse reactions induced by vaccines included in the national program of immunization for the first year of life. Method: Eighty-one children born between 1st of January 2018 and 31st of March 2019 were included. The vaccination refusal rate, and incidence and severity of adverse effects induced by three mandatory vaccines (Hexavalent, Prevenar 13 and MMR) were analyzed. The level of parents’ education and the sources of information that were consulted in order to understand the adverse effects were also analyzed. Results and conclusions: The rate of adverse events was lower than 30% of the total number of doses, and most of them were mild. The incidence and severity of vaccine-induced adverse effects were correlated with prematurity. The vaccine hesitancy rate was lower than the national one, possibly due to a high level of education and good information provided by doctors that might have led to parents’ concerns regarding vaccination being answered.

## 1. Introduction

Vaccines represent now the most important achievement of public health in the twentieth century. The widespread vaccination program led to the eradication of smallpox in the entire world, while polio is now diagnosed in only a few countries [1]. Despite these excellent results, in the last decade several European countries, including Romania, have gone through different outbreaks of vaccine-preventable diseases. Nowadays outbreaks are a consequence of the insufficient coverage of vaccinations that should attend to more than 95% of the eligible population to be efficient in preventing infectious diseases [1]. A descendent evolution of vaccination coverage in the last decade in most of the country is a result of vaccine hesitancy, which is considered a top ten global threat to public health by the World Health Organization [2,3].

Vaccine hesitancy is defined as a rejection or delay in vaccination despite the availability of efficacious vaccines [4]. Research in public health has focused on identifying the main factors that may explain parental vaccine hesitancy. The most frequent concerns among parents include the uncertainty of the usefulness and effectiveness of vaccination and questions about vaccine safety [5]. An extensive study conducted in 67 countries revealed that in European countries, a high level of distrust in vaccine safety is present, especially in France [6]. Healthcare providers could act as key components to improve the public’s trust by using scientific and epidemiological evidence. However, healthcare providers can be vaccine-hesitant themselves [7]. If family doctors do not trust the safety and efficacy of vaccines, then they are unlikely to convince patients or relatives to accept vaccines for their children. If the GP is vaccine-hesitant the communication between him/her and the parents is missing, and this lack of discussion may lead to an augmentation of the parents’ distrust. In order to find more information the parents may use other sources of information, such as social media. This attitude was shown clearly in the eastern part of Europe during the SARS-CoV-2 vaccination campaign in particular. The attitude and knowledge of GP’s about vaccines in general can influence their intention to recommend vaccination to their patients [8].

Socio-demographic factors associated with parental vaccine hesitancy vary across locations and temporal contexts [5]. Some studies have shown that younger adults and people with a lower level of education are more skeptical and have a negative attitude towards vaccination [9], while others have shown no correlation between gender, age, education and a positive attitude towards vaccination [10]. The European Centre for Disease Prevention and Control [11] has reported that social media is one of the main factors that increases vaccine hesitancy due to the rapid and widespread communication of rumors and misunderstandings regarding vaccination. The experts in public health and the entire medical community should be more aware of the huge negative potential of social networks in sharing wrong information about vaccines and they should correct false statements using language that appeals to those who are undecided about vaccination [2]. An individualized approach tailored to a specific community will likely be most effective in addressing vaccine hesitancy [12].

Each country adopts a national program of immunization in order to provide an answer to the general and special needs of the community that is in accordance to ECDPC recommendations [11]. In Romania, the national program includes five vaccines administered within the first year of life [13]. Two vaccines are administered immediately after birth in the maternity ward: the Bacille Calmette-Guérin (BCG) vaccine and monovalent vaccine against hepatitis B. The other three vaccines are given by family doctors: the Hexavalent vaccines against diphtheria, tetanus, pertussis, poliomyelitis, hepatitis B virus (HBV) and Haemophilus influenzae type b (Hib) (DT2aP-HBV-IPV-Hib), and the 13-valent pneumococcal conjugate vaccine (PCV13), both administered at 2, 4 and 11 months, and the measles, mumps, and rubella (MMR) vaccine given at 1 year of age. In Romania, a supplementary dose of the MMR vaccine was introduced in the national program of immunization in 2016 due to rubella outbreaks [13]. More vaccines have been added to infant schedules, so it is preferable to use combined vaccines to increase the compliance rates in this age group by reducing the number of injections needed to deliver the vaccines [14]. Co-administration of different vaccines also raised concerns regarding lower safety. However, experts concluded that co-administration regimens are well tolerated, in general, with an increased risk of fever when the Hexavalent 13 and valent pneumococcal vaccines are given on the same day [14,15]. In the case of high fever after vaccination, paracetamol appears to be more effective in reducing it than ibuprofen [15].

The present study analyzed the immediate adverse reactions induced by vaccines administered within the first year of life, with regards to the type and severity of them. As a secondary objective, the authors also analyzed the sources of information used by parents to understand and to manage the vaccine-induced adverse reactions, in order to identify factors that could be associated with vaccine hesitancy. Additionally, the rate of refusal was assessed.

## 2. Materials and Methods

### 2.1. Patients and Study Design

The study was observational, analytic, transversal, type. The present research analyzed data related to vaccination during the first year of life. Eighty-one children born between 1 January 2018 and 31 March 2019 were included in the present study. All the children were under surveillance of a single family doctor in Cluj Napoca, Romania. The sex ratio was F:M = 0.65. The study protocol was approved by the Ethics Committee of the University of Medicine and Pharmacy (nr. 283/28.07.2017), according to the principles from Declaration of Helsinki. All the parents accepted to sign the informed consent before inclusion, so no exclusion criteria were available for the present study.

The inclusion criteria were: children born in the aforementioned period, even if their parents refused the mandatory vaccines from national program of vaccination. All the children were evaluated during their 1st year of life. The evaluation was conducted at 2, 4, 9, 11 and 12 months of life when the mandatory vaccines are approved to be administered in Romanian national program of vaccination.

Romanian national program of vaccination included five mandatory vaccines in the first year of life. Two of them (monovalent antihepatitis B vaccine and BCG vaccine are administered immediately after birth in hospital settings) and the other 3 vaccines are administered by family doctor as follows:

- Hexavalent vaccine—against diphtheria-tetanus-pertussis-Hemophilus B-Hepatitis B-poliomyelitis—3 doses at 2, 4 and 11 months of life
- Prevenar 13—antipneumoccocal vaccine—3 doses at 2, 4 and 11 months of life
- MMR—mumps, measles and rubella—2 doses at 9 (supplementary dose) and 12 months. Due to measles epidemic in Romania in 2016, a supplementary dose of MMR vaccine was included in the national program of vaccination between 2016 and 2020 [13].

### 2.2. Data Collection

Some of the clinical data were collected from patients’ files:

1. Birth weight;
2. Type of birth (natural/vaginal or cesarian section);
3. Gestational age (term birth ≥ 37 weeks of pregnancy or premature < 37 weeks of pregnancy);
4. Suffering at birth (respiratory, neurological or cardiologic distress).

The first visit to the family doctor was an information and counseling visit. Parents were informed about the efficacy and safety of vaccines included in Romanian national program of vaccination. The parents also received the public assessment report of vaccines from GP’s office. The vaccination procedure was scheduled after this first visit. Parents were also questioned at the first visit about:

- Level of education—middle school, high school or university;
- Sources of information regarding drugs in general and vaccines—healthcare providers, online reliable medical sources of social media (Facebook, parents groups, radio, TV).

After the administration of the vaccine, within 48 h, the parents received a call from doctor office to answer if the child presented adverse effects. If the answer was affirmative, the parents were counseled to write them down and to report in the next visit for the following dose of vaccine.

At each visit for vaccine administration, the doctor quantified the side effects that might have occurred after each administration of vaccines and asked the parents what was their attitude in managing them.

The assessment of adverse reactions induced by vaccination was conducted based on their incidence and severity mentioned in public assessment report of each vaccine. The authors based the severity of them on the following scale:

0 = no adverse reaction, no change in child behavior;

1 = mild—fever < 38 °C, irritability, agitation, interrupted sleep, local sensitivity at touch, altered eating behavior, all the changes lasted 24–48 h;

2 = moderate—fever, 38–39 °C, spasm of crying, local erythema and minor local endurance;

3 = severe—syncope, spasm of crying, fever > 39 °C, skin eruption, extended erythema, well-defined nodule at the site of administration;

4 = very severe—convulsions, paresis, anaphylactic reactions, fever > 39.5 °C.

The presence of a single criterion from a certain level of severity led to the inclusion of reactions in that level of severity.

After vaccine administration the child remained for surveillance at least 15 min. The nurse monitored the reactions and the child behavior after vaccination procedure. The necessity of drug administration to control the adverse reactions induced by vaccines was also assessed. During vaccines’ administration, the pain intensity was also evaluated. The pain induced by the medical act was assessed in 2 ways:

1. On a scale from 0 (no pain and crying) to 10 (severe pain, anxiety and severe crying).
2. Duration of the anxiety and crying:
  0 no reaction;
  1 mild crying, less than 5 min;
  2 moderate–severe crying, more than 5 min.

### 2.3. Statistical Analysis

The statistical analysis was performed using MedCalc Statistical Software version 19.0.3 (MedCalc Software bvba, Ostend, Belgium; https://www.medcalc.org; 2019, accessed on 1 July 2022). Data were labeled as nominal or continuous variables. The nominal variables were expressed as percentage. The normal distribution for continuous variable was conducted using the Kolmogorov-Smirnov test and they were expressed as mean and standard deviation. The adequate statistic tests according to data distribution were chosen. The differences were assessed between groups by Mann-Whitney test. The χ^2^ test was also used for data analysis. Level of statistical significance was set at *p* < 0.05.

## 3. Results

Eighty-one children were evaluated during their first year of life. Demographic data are presented in Table 1.

More patients were boys and their parents were living in an urban area, and in most of the cases, the parents had finished a university education. None of the children’s parents had a middle school education. There was an equal distribution regarding the type of delivery between natural (vaginal) delivery and cesarian section.

Parents collected the information regarding the vaccines’ potential to induce side effects from different sources. A significantly higher percentage of mothers who graduated university were informed by online sources than mothers who finished only high schools (90.2% vs. 9.8%, *p* = 0.016). The same differences were also noticed in the case of the fathers’ education, more people that graduated university were informed by online sources than those that graduated from high schools (92.2% vs. 7.8%, *p* < 0.001). There were no significant differences between the type of parents’ education and information taken from social media or vaccines’ public assessment reports. Both mothers and fathers with a higher level of education preferred to be informed by more than one source about vaccines and their risk of side effects.

### 3.1. Adverse Reactions Induced by Vaccination

Most of the parents signed the consent for vaccination. From the entire group, the parents of two children refused the vaccination with the Hexavalent vaccine at 2 months and three parents refused the following doses. The most refusals were counted for the MMR vaccine (eight refusals of initial dose at 9 months, and five refusals at 12 months). For the Prevenar 13 vaccine, the rate of refusal was similar to the first administration, without fluctuations.

The refusal rate was correlated with the number of sources of information, being statistically significantly higher in parents who preferred to be informed by social media than by public assessment reports of the vaccines (*p* = 0.02 for Hexavalent vaccine and *p* < 0.001 for Prevenar 13 and MMR vaccines). Parents of seventeen children consulted social media in order to be informed about vaccines. From this group of parents between 11% and 35% of them refused the vaccination, and the rate varies with the type of vaccine (the highest rate being observed in the case of the MMR vaccine)

The adverse reactions induced by vaccinations were followed along the first year of life and the reactions after the mandatory vaccines included in the national program of vaccination were counted. The percentage of adverse reactions after each dose of vaccine and their severity are presented in Table 2.

Most of the adverse reactions were counted after the second dose of the Hexavalent vaccine and of the Prevenar 13 vaccine, but in both cases there were mild reactions in the majority of cases. The severe adverse reactions were noticed after the first dose of the MMR vaccine. The incidence and the severity of adverse reactions were not correlated with patients‘ gender or with the type of delivery. The incidence of adverse reactions was higher in premature children, especially the reactions that occurred after the second and third doses of the Hexavalent vaccine (*p* = 0.019) and Prevenar 13 vaccine (*p* = 0.016)

**Figure 1:**
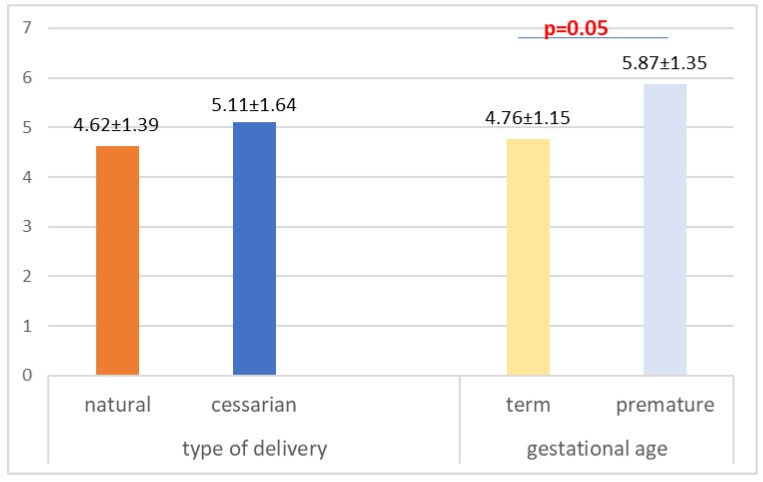
The intensity of pain during the vaccination procedure according to gestational age and type of delivery.

The pain induced by the vaccination procedure was evaluated based on child crying intensity, on a scale from 0 to 10. The intensity of pain at the vaccination moment was not influenced by the type of delivery (*p* = 0.14), but it was influenced by the gestational age (*p* = 0.05) (Figure 1). There is no correlation between the intensity of pain and the birth weight (R = −0.174, *p* = 0.12).

**Figure 2:**
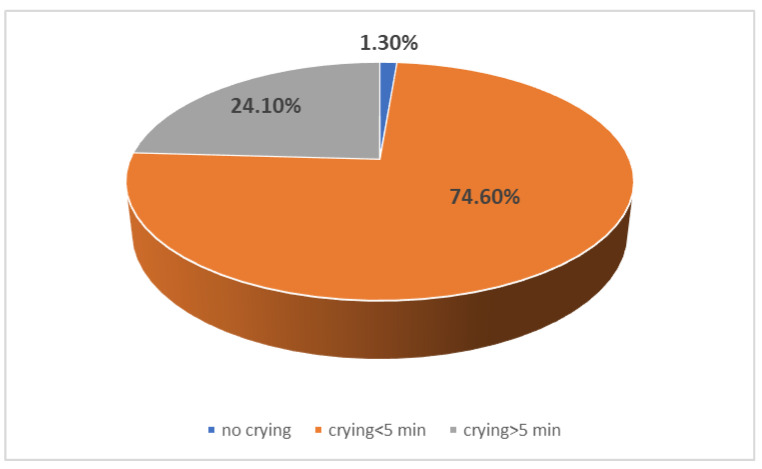
Distribution of patients according to duration of calming down.

The crying and anxiety after vaccination was also evaluated based on their duration (less or longer than 5 min). Most of the children had a short period of crying after vaccination (less than 5 min) and they were quickly calmed down (Figure 2). The duration required for calming down after vaccination was not correlated with child gender, gestational age, type of delivery, type of vaccination or the number of vaccine dose (*p* > 0.005).

### 3.2. Management of Adverse Reaction

The information regarding how to manage the adverse effects after vaccination could be obtained from different online sources, from vaccines’ public assessment reports or from medical healthcare providers. All of the parents required and obtained information from medical healthcare providers about how to manage the possible side effects after vaccine administration. More mothers with a higher level of education preferred to be informed about them from online sources (42.4% vs. 13.3%, *p* = 0.041) or from the vaccines’ public assessment reports (22.7% vs. 0%, *p* = 0.033) compared to those with a lower level of education. Regarding fathers’ information methods, we noticed that persons with a higher level of education more frequently used online sources (43.8% vs. 11.8%, *p* = 0.022) compared to those with a lower level of education, but no differences were noticed for information from the vaccines’ public assessment reports.

Both mothers and fathers with a higher level of education used an increased number of sources than those with a lower level of education (1.13 ± 0.35 vs. 1.65 ± 0.45, *p* = 0.011, respectively, 1.23 ± 0.43 vs. 1.64 ± 0.46, *p* = 0.04). There is no correlation between the number of sources used for information regarding side effects and the type of delivery or gestational age (*p* > 0.05). There is no correlation between the type of information or number of sources and the intensity of pain during the procedure (*p* > 0.05).

**Figure 3:**
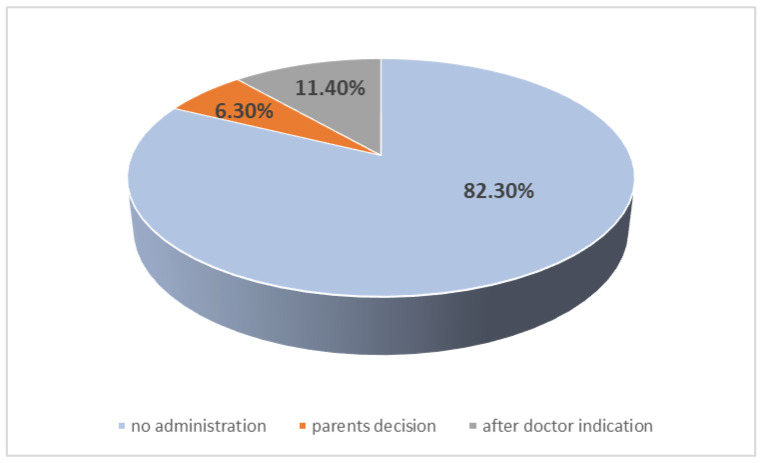
Administration of therapy after vaccines.

None of the parents gave analgesic and anti-inflammatory drugs such as paracetamol or ibuprofen before vaccine administration to prevent further side effects. Only 14 children required the administration of drugs to manage side effects: 5 of them (6.3%) received the treatment after the parents’ decision, while 9 (11.4%) of them after healthcare provider indication. The distribution of treatment administration is presented in Figure 3. There is no correlation between pain intensity during vaccination and the treatment recommended after vaccine administration (*p* > 0.05). Moreover, there is no correlation between the type of vaccine, the number of dose and the administration of medication after vaccine administration.

## 4. Discussion

The present observational study demonstrated that vaccines are a safe preventive method for infectious diseases in children below 1 year old. The refusal rate of vaccination was below the national level of the same year.

Vaccines represent one of the most important preventive strategies in paediatric populations. In the last decade, the concerns regarding vaccines safety have risen significantly and, in particular, parents are continuously questioning the need for and the safety of vaccines. Therefore, the vaccination rates have fallen to a suboptimal level in some countries and communities [16]. The present study reflects that healthcare providers that work in primary care are well trained in vaccine administration. They informed correctly all the children’s parents regarding the necessity of vaccination and their safety.

Despite the fact that family doctors provide qualified information and give parents the opportunity to ask questions about both the benefits of vaccination and possible side effects, more than half of the patient also prefer to be informed from virtual sources, including social media, and because of this, their decisions can be negatively influenced. Bianco et al. [9] reported that 7.7% of subjects were defined as vaccine-hesitant parents based on a questionnaire score, while 24.6% refused or delayed at least one dose of vaccine for their child. These negative decisions were commonly noticed in those parents who received information from the mass media. It is known that a negative media may influence the decision-making process of vaccination. Vaccine skepticism increased after the publication of Wakefield’s article in The Lancet [17], which was potentially made possible by the increased negative media coverage of MMR [16]. This information may support a campaign for positive vaccination in the mass media, with a clear positive message for people with a higher level of education who decide to be well informed from different sources. The refusal rate was correlated with the number of information sources and the type of them, confirming the previously published data. In the present study, parents who refused the vaccination were those who were informed about them from different sources, including social media, which may contribute to a negative attitude regarding vaccines. A study published in 2017, based on the responses of parents of 6-year-old children, reported that those with positive attitudes for vaccines were more likely to report physicians as their source of information about vaccines rather than social media [10]. Even if the parents who refused the vaccines were informed at the beginning by GPs, consulting multiple sources of information, which contain contradictory data, can lead to an increase in parents’ mistrust. In 50% of the cases in which the first administration of the MMR vaccine was refused, the parents did not read the information about the vaccine and preferred to only be informed by social media. However, further discussion with a GP led to an acceptance rate for the regular dose of the MMR vaccine at 12 months. This confirms that a positive attitude of the GP coupled with several meetings and discussions with parents may increase the rate of vaccination.

However, almost a quarter of parents decided to read and to discuss with the family doctor the information included in the public assessment reports, which means they accepted that vaccines, like all drugs, may induce side effects, which are mild in most cases. The role of health care providers in vaccination counseling is very important for the decision-making process [18,19]. A previous study that analyzed information in several European Countries reported that doctors, especially those from primary care, and pharmacists are considered the most trustworthy sources of information about medicines [20]. Moreover, in the present study, all the parents were informed about the vaccines’ efficacy and safety, confirming that health care providers represent the main pillar of vaccination literacy.

In the present study, the vaccine refusal rate was lower than the national rate estimated in 2018 [13,21]. The refusal rate for the Hexavalent vaccine was 2.5% for the first dose and 3.7% for the next ones, significantly lower compared to the national rate, which was estimated to be between 15 and 20% [21]. Higher rates of refusal were noticed for the Prevenar 13 and MMR vaccines. The main reason for refusing the Prevenar 13 vaccine was the fact that it was recently introduced in the national schedule of vaccination. Regarding the MMR vaccine, the main concern was its safety and the risk of autism. The refusal of the MMR vaccine due to the risk of autism appeared after the year 2000 with the “Wakefield Incident” [17]. Although later there were more than 25 studies that showed no link between the MMR vaccine and autism [22,23,24] and the article published in The Lancet was retracted [25], the impact of the study published in 1998 by the journal led to a significant decline in measles and rubella vaccination coverage worldwide [26], not only in Romania.

Actually, due to a rubella epidemic declared in Romania in 2016, the national program of immunization was changed, to include one dose of the vaccine at 9 months and the second one at 12 months. The refusal rate was almost 10% for the first administration, but for three children there was actually a delay of the first administration up to 12 months. The Romanian vaccination rate for MMR was 89.6% for the first dose and 80.9% for the second one, in 2018. In this study, the authors reported a higher rate of vaccination for the second administration at 12 months, but for three children, the parents were counseled to allow the vaccination even if the first dose was delayed. It seems that a repeated discussion with parents may influence their positive decision for vaccination. In other countries, the vaccination rate for MMR is lower than what was reported in Romania and in the present study and it has a descendent evolution. For example, in Southern Italy, the vaccination rate for MMR in 2016 was 69.6% in children < 2 years old [27]. On the contrary, in Germany, vaccination coverage for the first dose of the MMR vaccine in children <15 months of age increased significantly from 83% in 2008 to 89.5% in 2014 [28], a rate that is close to the Romanian one in 2018. On the other hand, the study was conducted in an urban area and the parents had access to several sources of information, which might explain a higher coverage through vaccination than that in the present research. Another explanation could reside in the fact that Romania reported the most cases of rubella and the epidemic conditions were considered until 2020, meaning that parents were afraid for their children and accepted the vaccination more easily, especially in urban areas where easy access to health care providers exists. The positive attitude of the GPs towards vaccination and a good communication with parents may also explain a higher rate of vaccination in this center vs. the national rates.

Even if the rate of side effects induced by vaccine administration was almost 30%, less than 2% were actually moderate and severe adverse events. Most of the side effects occurred after the second or the third dose of the Hexavalent and Prevenar vaccines and they were mild. In a French study published in 2017 [29], the authors analyzed all the adverse events related to Prevenar 13 administration. They counted 376 severe adverse events in a period of surveillance of 14 years, but most of them were related to the administration of Prevenar in association with other vaccines, usually the Hexavalent vaccine [29], similar to the present study. The authors raised the hypothesis that the severity of adverse reactions could be determined by the concomitant administration of two vaccines. Most of the adverse reactions were cutaneous or fever [29], as in the present study. The same observations were noticed in a Spanish review of Prevenar 13 safety [30]. They reported only mild and moderate adverse effects related to both Prevenar 7 and Prevenar 13 administration [30]. In another meta-analysis that included safety data from 13 studies conducted in 9 different countries from Europe, North America and Asia, the authors reported similar rates of local side effects to those in the present research, concluding that they were the types of conditions and symptoms expected in infants and children [31].

The severe side effects (0.46%), consisting of fever >39 °C, skin eruption and extended erythema, were noticed at the first administration of the MMR vaccine. It was an expected result considering that the MMR vaccine is a live attenuated one, compared to Hexavalent and Prevenar-13, which are inactivated vaccines. The incidence of adverse effects induced by MMR was in the range reported in the public assessment report of the vaccine. Indeed, other research also reported an increased risk of side effects after the MMR vaccine. Gidengil et al. [32] analyzed in a recently published systematic review, the safety of vaccines recommended for children. There was no evidence of an increased risk of severe adverse events after most of the vaccines administered in childhood, except for an increased risk of febrile seizures with MMR. Similarly, Yamoah et al. [33] reported a low risk of the association of severe adverse events with vaccines used in routine immunization in Africa, concluding that a strong association of severe adverse events with the BCG vaccine may be noticed [33]. In the present study, the authors evaluated only the immediate adverse event related to vaccination not the long-term risk. All the children were monitored for 1 year and the doctors counted side effects that might have occurred immediately or within 72 h after vaccine administration. Severe fever over 39 °C was noticed only in the case of MMR administration, but none of the children developed fever-induced seizures. All these children received the appropriate treatment for fever in order to prevent seizures’ occurrence and that which the family doctor recommended: it was not a self-decision of the parents based on their information from online sources. In none of the cases were other unknown side effects or adverse reactions reported that had an unknown frequency induced by the MMR vaccine. As we previously presented, most of the side effects were present after the second or third dose of vaccine, different from other vaccines, such as anti-SARS-CoV-2 in which the incidence of adverse events was higher after the first dose [34]. Thus, the severity of vaccine-induced adverse event depends not only on the type of vaccine but also on the patients’ age and immune status. All of the children included in the present analysis were in good health, without any immune deficiency, so the expected immune response was a good one. This is another reason for why a low rate of severe adverse effects after vaccination was noticed.

The severity of pain evaluated by the intensity of crying and anxiety in the moment of vaccination and the incidence of side effects induced by vaccines were higher in preterm children. Ramsay et al. [35] reported no difference in the incidence and severity of adverse effects within 12 h after immunization between term and preterm infants, but they evaluated trivalent vaccines (diphtheria-tetanus-pertussis). In the present research other vaccines were evaluated that were more complex with several microbial strains included, and some of them were administered together in a single visit. Wilck et al. [36] evaluated the incidence of adverse effects after the Hexavalent vaccine, reporting that the incidence and severity of crying and fever as adverse events was comparable between the general population and premature infants and between term and preterm infants. Local and systemic reactions induced by the antipneumoccocal vaccine were also similar for both term and preterm populations [37]. Although moderate/severe adverse reactions have been associated with prematurity in the present research, the reactions were manageable with minimal surveillance and treatment. We may conclude that the regular schedule for immunization should be followed in both term and preterm infants as Chiappini E et al. suggested [38]. The health care provider should discuss these aspects with parents, confirming that prematurity is not a contraindication or a reason to delay immunization in spite of more severe pain in the moment of vaccine administration.

The main strength of this paper is that it evaluates the safety of immunization in infants within the first year of life, correlated with parents’ source of information. The study showed that the safety of vaccines is well known, the existence of adverse reactions are known among parents and they accept the counseling of health care providers in order to be informed about vaccination. Good pre-vaccination information of parents may increase their confidence of the vaccines in immunization and this could be one of the strategies to improve the vaccination rate. Parents are aware of the existence of adverse reactions and under a doctor’s surveillance they can safely manage the occurrence of them. There are also a few limitations in this study. Firstly, a small number of patients were included in the study. Exploring vaccine hesitancy and information sources in a larger sample size may be useful to understand attitudes towards refusal of specific vaccines. Secondly, there is no correlation with other centers, for example, from rural areas, to analyze parents’ beliefs and knowledge about vaccination in a deeper way.

## 5. Conclusions

The rate of side effects’ occurrence was lower than 30% of the total number of doses, and most of them were mild. The incidence and severity of vaccine-induced adverse effects was correlated with prematurity but not with other demographic or clinical data. No adverse effects with unknown frequency or severity induced by vaccines were reported. The rate of vaccine refusal was also low in the present study, being correlated with the number of sources of information consulted by parents and with the type of vaccine. The positive attitudes of GPs towards vaccination and repetitive discussions with parents may increase the rate of vaccination. The results should be examined with caution due to the relatively small number of subjects.

## Figures and Tables

**Table 1:** Demographic data.

Parameter		Distribution (n, %)
Gender	M	49 (60.5%)
	F	32 (39.5%)
Living aria	Urban	66 (81.5%)
	Rural	15 (18.5%)
Type of delivery	Vaginal	41 (50.6%)
	Cesarian	40 (49.4%)
Parents’ studies		
Mother	University	66 (81.5%)
	High school	15 (18.5%)
	Middle school	0
Father	University	64 (21%)
	High school	17 (79%)
	Middle school	0

**Table 2:** Severity of adverse reactions induced by vaccine administration.

Vaccine	Severity of Adverse Reactions				
	No AD	Mild	Moderate	Severe	Refusal
Hexa 2 months	60 (74.1%)	17 (20.9%)	2 (2.5%)	0	2 (2.5%)
Hexa 4 months	43 (53.1%)	34 (42%)	1 (1.2)	0	3 (3.7%)
Hexa 11 month	46 (56.8%)	30 (37%)	2 (2.5%)	0	3 (3.7%)
Prevenar 13, 2 months	59 (72.7%)	16 (19.8%)	2 (2.5%)	0	4 (5%)
Prevenar 13, 4 months	44 (54.3%)	32 (39.5%)	1 (1.2%)	0	4 (5%)
Prevenar 13, 11 months	46 (56.8%)	29 (35.7%)	2 (2.5%)	0	4 (5%)
MMR 9 months	65 (80.2%)	5 (6.2%)	0	3 (3.7%)	8 (9.9%)
MMR 12 months	72 (88.8%)	4 (5%)	0	0	5 (6.2%)

Abbreviations: Hexa, Hexavalent vaccine; MMR, mumps, measles and rubella.

## Data Availability

The data presented in this study are available on request from the corresponding author. The data are not publicly available due to privacy and ethical restrictions.
